# Task Sharing in Neurosurgical Care Under Japan’s Work-Style Reform: A Collaboration With Nursing Designated Care (NDC) in Subarachnoid Hemorrhage Management

**DOI:** 10.7759/cureus.94744

**Published:** 2025-10-16

**Authors:** Taigen Sase, Homare Nakamura, Yukiko Nakamura, Kiyotaka Wakatsuki, Gaku Hidaka, Hidetoshi Murata

**Affiliations:** 1 Department of Neurosurgery, St. Marianna University School of Medicine, Yokohama Seibu Hospital, Yokohama, JPN; 2 Department of Nursing, St. Marianna University School of Medicine Yokohama Seibu Hospital, Yokohama, JPN; 3 Department of Neurosurgery, St. Marianna University School of Medicine, Kawasaki, JPN

**Keywords:** nurse practitioner (np), nursing designated care, subarachnoid hemorrhage, task shifting, work-style reform

## Abstract

Beginning in April 2024, work-style reforms were implemented for doctors. We analyzed the changes in our medical practice resulting from the assignment of a designated care nurse, called nursing designated care (NDC), to the Department of Neurosurgery, in line with work-style reforms. This study aimed to evaluate whether introducing an NDC into a neurosurgical department could redistribute perioperative tasks and improve workflow efficiency in subarachnoid hemorrhage (SAH) management under Japan’s physician work-style reforms. We compared medical care over a one-year period from April 2022, when our department had three neurosurgeons, and over a one-year period from April 2023, under a new system in a four-member team consisting of three neurosurgeons and one NDC. The target disease was a SAH. We compared medical care from initial treatment in the emergency room (ER) to the operating room, medical care from leaving the operating room to postoperative management after returning to the intensive care unit, and management of the cerebral vasospasm phase. Some of the work that had been performed by the neurosurgeons was taken over by the NDC, and the duties of the neurosurgeons were shared. Clinical intervention by the NDC in the ER for patients with SAH may contribute to shortening the treatment time from the start of treatment in the ER to admission in the operating room. In the management of the cerebral vasospasm phase, the NDC was able to intervene with multiple specified acts, and the neurosurgeon's duties were shared. We introduced our department's current treatment methods in line with the work-style reforms for doctors. Through collaboration with the NDC, it may be possible to divide work in line with the work-style reforms for neurosurgeons.

## Introduction

Since April 2024, work-style reforms have been applied to doctors in Japan. According to the Ministry of Health, Labor and Welfare, these reforms were introduced in response to increasing burdens on individual physicians, driven by changing medical needs, the increasing complexity of medical care, and a decline in the medical workforce. Prior to these reforms, approximately 50% of full-time hospital physicians were working more than 960 hours of overtime annually. Neurosurgeons, along with emergency physicians, obstetricians-gynecologists, and surgeons, are particularly prone to long working hours. To maintain the quality of care while complying with these regulations, new management systems are required.

Nurses qualified to perform specified acts (38 procedures in 21 categories) are collectively referred to as nursing designated care (NDC) nurses and have attracted attention as potential contributors to task shifting in Japan. Specified acts are performed under the comprehensive direction of a supervising physician, and many are related to neurosurgery. NDC is a nursing qualification unique to Japan, introduced in 2015. Therefore, there is a growing interest in the entry of NDC into the clinical field [1]. On the other hand, Japanese nurse practitioners (JNPs), who completed graduate NP programs, can perform all 38 specified acts and additional procedures under direct physician supervision. Previous studies have demonstrated that task shifting to JNPs effectively reduces physicians’ workload [2-7].

Since April 2023, an NDC has been part of our department, collaborating in the care of patients with subarachnoid hemorrhage (SAH). In this study, we describe our treatment model incorporating NDC collaboration and evaluate its impact on task sharing and workflow efficiency, with particular attention to the collaboration between neurosurgeons and NDCs and the transfer time from the emergency room (ER) to the operating room (OR) or intensive care unit (ICU).

## Materials and methods

Our hospital is a university-affiliated branch hospital with a tertiary critical care center located in the western part of Yokohama City. Since the hospital opened, the number of full-time neurosurgeons has fluctuated between five and six; however, from October 2021 to March 2024, there were three.

When the first author returned in April 2023, one NDC was assigned to the department of neurosurgery simultaneously. The NDC assigned to our department is not a JNP, but an NDC who completed 21 categories and 38 procedures curricula (Table 1) provided by designated nongraduate training institutions. The differences from JNP are discussed in the Discussion section.

**Table 1:** 21 categories and 38 specific acts Many procedures are related to neurosurgery

21 categories		38 specific acts	
no.		no.	
1	Respiratory system (related to airway management)	1	Adjustment of the position of oral or nasal tracheal tubes
2	Respiratory system (related to artificial respiratory therapy)	2	Setting change of invasive positive pressure ventilation
		3	Setting change of non-invasive positive pressure ventilation
		4	Adjustment of sedative dosage for patients on artificial respiration
		5	Weaning from artificial respiration
3	Respiratory system (related to long-term respiratory therapy)	6	Replacement of tracheal cannula
4	Circulatory system	7	Operation and management of temporary pacemaker
		8	Removal of temporary pacemaker lead
		9	Operation and management of percutaneous cardiopulmonary support device
		10	Adjustment of frequency of support when weaning from intra-aortic balloon pumping
5	Pericardial drain management	11	Removal of pericardial drain
6	Chest drain management	12	Setting and changing the suction pressure of a low-pressure continuous thoracic suction device
		13	Removal of chest drain
7	Peritoneal drain management-related	14	Removal of a peritoneal drain (including removal of a puncture needle placed in the abdominal cavity)
8	Fistula management	15	Replacement of a gastrostomy catheter or enterostomy catheter or gastrostomy button
		16	Replacement of a bladder fistula catheter
9	Nutrition-related catheter management (central venous catheter)	17	Removal of central venous catheter
10	Nutrition-related catheter management (peripherally inserted central venous catheter)	18	Insertion of peripherally inserted central venous catheter
11	Wound management	19	Removal of necrotic tissue with no blood flow in the healing of pressure ulcers or chronic wounds
		20	Negative pressure closure therapy for wounds
12	Wound drain management	21	Removal of wound drain
13	Arterial blood gas analysis	22	Blood sampling by direct arterial puncture
		23	Insertion of radial artery line
14	Dialysis management	24	Management of hemodialysis or hemodiafiltration in acute blood purification therapy
15	Drug administration related to nutrition and hydration management	25	Adjustment of high-calorie infusion during continuous infusion
		26	Correction of dehydration symptoms with infusion
16	Drug administration related to infection	27	Temporary administration of drugs to patient with signs of infection
17	Drug administration related to blood glucose control	28	Adjustment of insulin dosage
18	Postoperative pain management	29	Administration of analgesics through epidural catheter and adjustment of dosage
19	Drug administration related to circulation	30	Adjustment of catecholamine dosage during continuous infusion
		31	Adjustment of sodium, potassium, or chloride dosage during continuous infusion
		32	Adjustment of antihypertensive drug dosage during continuous infusion
		33	Adjustment of glucose or electrolyte infusion dosage during continuous infusion
		34	Adjustment of diuretic dosage during continuous infusion
20	Drug administration related to mental and neurological symptoms	35	Temporary administration of anticonvulsants
		36	Temporary administration of antipsychotics
		37	Temporary administration of antianxietics
21	Drug administration related to skin damage	38	Local injection and dosage adjustment of steroid drugs when anticancer drugs or other drugs leak out of blood vessels

The NDC in our department works weekday daytime shifts (8:30-17:00) with no night shifts. Regulations regarding holidays are in accordance with hospital regulations and are the responsibility of the nursing department. Reports on collaboration with NDCs remain scarce, underscoring the originality of this methodology [1].

We targeted the period from April 2022 to March 2023 (period A), when there were three full-time neurosurgeons, and the period from April 2023 to March 2024 (period B), when three full-time neurosurgeons and one NDC were assigned. During both periods, there were six slots per week for general outpatient clinics in the morning from Monday to Saturday (closed on the first and third Saturdays), and each neurosurgeon was in charge of two slots. In addition, each neurosurgeon was away from the hospital for one research day on a single weekday.

The general clinical flow of SAH management is summarized in Tables 2, 3. Depending on the situation, patients may be temporarily admitted from the emergency primary care room to the ICU ward. Our hospital is a tertiary critical care emergency center that often treats severe cases, and the initial treatment is mostly performed by emergency physicians. Since many cases are severe, endotracheal intubation is often performed to secure the airway prior to diagnosis.

**Table 2:** Clinical flow of period A CT: computed tomography; CTA: computed tomography angiography; CVC: central venous catheter; ICU: intensive care unit; SAH: subarachnoid hemorrhage The clinical flow of Period A is shown

Time course	Medical examination place	Doctor in charge	Content of medical procedure
↓	Emergency room	Emergency physician	Primary treatment after ambulance arrival
↓	(move)		Accompanying the patient
↓	CT room		CT/CTA (diagnosed SAH)
↓	Primary treatment contacts a neurosurgeon		
↓	CT room	Neurosurgeon	diagnosed ruptured lesion, and coordinating with an anesthesiologist and nurses in an operating room
↓	(move)		Accompanying the patient
↓	X-ray room		X-ray examination
↓	(move)		Accompanying the patient
↓	Emergency room		Adjusting the respiratory system and initiating sedation, analgesia, antihypertensive therapy, and so on
↓	Explanation room		Preoperative explanation for the family (patient's condition and various consent forms
↓	Emergency room		Initial treatment continuation and electronic medical record work
↓	(move)		Accompanying the patient
↓	Operating room or angiography room		Preparation for surgery and start of surgery
↓	Surgical treatment (craniotomy or endovascular)		
↓	Operating room or angiography room	Neurosurgeon	Finished surgery
↓	(move)		Accompanying the patient
↓	CT room		Checked the postoperative CT
↓	(move)		Accompanying the patient
↓	X-ray room		Checked the postoperative X-ray
↓	(move)		Accompanying the patient
↓	ICU		Postoperative medical care
↓	Explanation room at the ICU		Postoperative explanation for the family
↓	Family visitation to the patient		
↓	ICU	Neurosurgeon	Inserting a CVC and finishing postoperative care

**Table 3:** Clinical flow of period B CT: computed tomography; CTA: computed tomography angiography; CVC: central venous catheter; ICU: intensive care unit; SAH: subarachnoid hemorrhage; NDC: nursing designated care; PICC: peripherally inserted central venous catheter The clinical flow of period B is shown. Some of the tasks previously performed by neurosurgeons are now being performed by nursing designated care

Time Course	Medical examination place	Doctor in charge	Doctor's content of medical procedure	NDC	NDC's content of medical procedure	Task shifting and sharing
↓	Emergency room	Emergency physician	Primary treatment after ambulance arrival			
↓	(move)		Accompanying the patient			
↓	CT room		CT/CTA (diagnosed SAH)			
↓	Emergency physician contacts a neurosurgeon. The neurosurgeon contacts an NDC to request assistance					
↓	CT room	Neurosurgeon	Diagnosed a ruptured lesion, and coordinating with an anesthesiologist and nurses in the operating room	NDC	NDC shares the neurosurgeon's management plan and receives comprehensive instructions	Task sharing
↓	(move)	Task shifting from neurosurgeons to NDC			Accompanying the patient (Table 1, specified act category no. 2)	Task shifting
↓	X-ray room				X-ray examination	
↓	(move)				Accompanying the patient (Table 1, specified act category no. 2)	
↓	Emergency room				Adjusting the respiratory system and initiating sedation, analgesia, antihypertensive therapy, and so on (Table 1, specified act category no. 2, and 19)	Task sharing
↓	Explanation room	Neurosurgeon	Preoperative explanation for the family (patient's condition and surgical procedure, only)			
↓	Emergency room		Initial treatment continuation and electronic medical record work		Obtained various consent forms (consent for blood transfusion, consent for plasma fractionation products, consent for physical restraint, consent for peripheral central venous catheter (PICC) placement, etc.)	
↓	(move)	Task shifting from neurosurgeons to NDC			Accompanying the patient (Table 1, specified act category no. 2)	Task shifting
↓	Operating room or angiography room	Neurosurgeon	Preparation for surgery and start of surgery			
↓	Surgical treatment (craniotomy or endovascular)					
↓	Operating room or angiography room	Neurosurgeon	Finished surgery			
↓	(move)		Accompanying the patient			
↓	CT room		Checked the postoperative CT	NDC	NDC shares the neurosurgeon's postoperative management plan and receives comprehensive instructions	Task sharing
↓	(move)	Task shifting from neurosurgeons to NDC			Accompanying the patient (Table 1, specified act category no. 2)	Task shifting
↓	X-ray room				Checked the postoperative X-ray	
↓	(move)				Accompanying the patient (Table 1, specified act category no. 2)	
↓	ICU				Postoperative care in ICU, including PICC insertion (Table 1, specified act category no. 2, 10, 13, 15, 16, 17, 19, and 20)	Task sharing
↓	Explanation room at ICU	Neurosurgeon	Postoperative explanation for the family			
↓	ICU		Postoperative medical care			
↓	Family visitation to the patient					

Regarding the management of the cerebral vasospasm phase, management during period A was centered on fasudil hydrochloride, while in period B, management shifted from fasudil hydrochloride to clazosentan hydrochloride. In cases using clazosentan during period B, the cerebral vasospasm phase was managed mainly based on the parameters listed in Table 4. There are many intervention points for NDC, such as inserting peripherally inserted central venous catheter (PICCs) and adjusting fluid intake volume and diuretics (Table 1, specified act category nos. 10, 15, and 19). Other interventions by the NDC include many specified acts necessary for critical neurosurgery management, such as adjusting antibiotics, ventricular drain management, and antiepileptic drug management (Table 1, specified act category nos. 12, 16, and 20).

**Table 4:** Management parameters of the cerebral vasospasm phase CVP: central venous pressure; IVC: inferior vena cava; NDC: nursing designated care; PICC: peripherally inserted central venous catheter There are many intervention points for nursing designated care, such as inserting peripherally inserted central venous catheters and adjusting fluid intake volume and diuretics

Parameter	Staff in charge	Content of medical procedure
Body weight	General nurses	・Minimally invasive procedure ・Burden on nurses
Vital signs	General nurses	・Minimally invasive procedure ・Regular nursing duties
Fluid infusion volume and urine output	General nurses	・Minimally invasive procedure ・Regular nursing duties
CVP	General nurses	・Measurement with PICC ・PICC insertion by NDC (Table 1, specified act category no. 10)
IVC	NDC	・Ultrasonic examination by NDC
Chest X-ray	Radiographer	・X-ray request by doctor ・The request can be made on behalf of NDC
Others	Others	others
Comprehensive judgment	Neurosurgeon	・Neurosurgeon provides comprehensive instructions for the NDC. ・There are many intervention points by NDC, such as inserting PICCs and adjusting fluid intake volume and diuretics (Table 1, specified act category no. 10, 15, and 19).

However, the NDC only works during the day and assists with all work in the general neurosurgery, critical care, and emergency outpatient wards. Therefore, the NDC is often unable to be involved in any of the processes shown in Table 3.

The three neurosurgeons in periods A and B were different. All three neurosurgeons were certified specialists by the Japan Neurosurgical Society during each period, but each had a unique way of working. In addition, there are nonclinical duties within the hospital depending on the doctor's position. Therefore, because working hours are likely to vary from doctor to doctor, overtime hours were not assessed.

Consecutive patients with aneurysmal SAH admitted between April 2022 and March 2024 were screened. A total of 28 patients met the criteria: 13 in period A and 15 in period B. Clinical information and timing metrics were obtained by manual chart review of the electronic medical record by the first author. The start of treatment was defined as the ER arrival time documented at triage. Arrival to OR/ICU was defined as the official admission timestamp recorded in the electronic chart. The outcome was ER-to-OR/ICU transfer time (minutes). Observations included the type of NDC-performed specified acts and qualitative changes in task distribution during perioperative and vasospasm-phase management. Student’s t-test compared mean ER-to-OR/ICU times between periods. Given the small sample size, results are interpreted as exploratory; no power calculation was feasible.

## Results

Some of the tasks previously performed exclusively by neurosurgeons were reallocated to the NDC during period B, resulting in partial task shifting. Although the NDC worked only daytime shifts, their involvement still reduced the direct burden on neurosurgeons.

NDC participation in ER care for SAH may contribute to shortening treatment times (Table 5). In the management of the cerebral vasospasm phase, NDCs performed multiple specified acts, further sharing the neurosurgeons’ workload. The outcome was ER-to-OR/ICU transfer time. Mean times were 164.8 minutes (period A) versus 172.3 minutes (period B) with no statistically significant difference (p = 0.8). The shortest daytime ER-to-OR time improved from 189 min (period A) to 127 min (period B).

**Table 5:** Summary of results ER: emergency room; ICU: intensive care unit; OR: operating room Values are presented as means unless otherwise indicated. “Period A” represents April 2022-March 2023 (three neurosurgeons), and “period B” represents April 2023-March 2024 (three neurosurgeons and one NDC). The p-value was calculated using Student’s t-test

Period	Number of patients (n)	Mean time ER→ICU/OR (min)	p-value	Shortest time to OR during daytime (min)
A	13	164.8	0.8	189
B	15	172.3		127

## Discussion

Work-style reforms have been applied to doctors since April 2024 in Japan. These reforms aim to reduce excessive working hours and improve physician well-being but present challenges for maintaining high-quality care. In neurosurgery, the perioperative management of SAH is particularly labor-intensive, necessitating new strategies for task sharing.

NDCs, nurses trained to perform specified acts, are increasingly recognized as key collaborators in multidisciplinary care [1]. JNPs are NDCs in a broad sense because they can perform all 38 specified acts. However, because JNPs can perform procedures under the direct direction of a doctor in addition to the 38 specified acts, they are often distinguished from NDCs. NDCs, in a narrow sense, excluding JNPs, can selectively undergo specific practice training for one specified act. Therefore, there are NDCs who can perform all 38 specified acts and NDCs who can only perform some of the specified acts. According to the Ministry of Health, Labor and Welfare, as of August 2023 in Japan, 8,802 nurses have completed training in specified acts (NDCs in the broad sense, including JNPs). The Japanese Organization for Nurse Practitioner Faculties announced that the total number of JNPs was 872 as of April 2024. In addition, the exact number of NDCs able to perform all 38 specified acts has not been made public, but it is estimated to be less than that of JNPs [1].

Assigning an NDC to our department allowed a reallocation of several perioperative tasks from neurosurgeons to nursing staff. Although NDC participation coincided with a shorter minimum daytime ER-to-OR time (189 → 127 min), the overall mean time did not improve significantly. Therefore, efficiency-related findings should be interpreted as preliminary observations that require confirmation in larger controlled studies. 

Our previous questionnaire survey confirmed reduced burden for physicians and ward staff [1]. Previous studies have demonstrated that task shifting to JNPs effectively reduces physicians’ workload, particularly in surgical and perioperative settings [2,4-7]. Katayama et al. reported improved working environments for neurosurgeons after the introduction of NPs [6], and similar benefits have been observed in neuroendovascular therapy [7].

International systematic reviews and randomized trials have consistently demonstrated that NP-led care achieves comparable clinical outcomes, high patient satisfaction, and cost-effectiveness [8-12]. McDonnell et al. evaluated the implementation of advanced nurse practitioner (ANP) roles in acute hospital settings and confirmed their positive impact on quality of care [8]. Mundinger et al. demonstrated in a randomized trial that patient outcomes under nurse practitioner (NP)-led primary care were equivalent to those under physician-led care [9]. Stanik-Hutt et al. reported in a large systematic review that NP-led care and physician-led care have equivalent outcomes [10]. Newhouse et al. confirmed that NP practice is associated with favorable clinical outcomes and improved cost-effectiveness [11]. Furthermore, Swan et al. reported that the introduction of NPs improves patient satisfaction and the overall quality of care [12]. Our study expands on this body of evidence by providing a detailed description of task redistribution in the perioperative management of SAH in a Japanese tertiary neurosurgical center.

Despite these benefits, several considerations remain regarding the introduction of NDCs into clinical practice. There is a potential risk that task sharing with NDCs could delay the acquisition of patient management skills among interns and residents who care for critically ill patients. Nevertheless, previous studies have reported that NPs can coexist with junior trainees, taking a leadership role in procedures such as PICC insertion without depriving them of opportunities to perform medical procedures [4]. Another challenge concerns the employment status and salary structure of NDCs, which remain equivalent to those of general nurses. As reported previously [13], insufficient compensation has been associated with decreased motivation and job turnover among NDCs, suggesting that improving working conditions is essential for long-term sustainability.

Study limitations

This study has several limitations. All neurosurgeons during the study period were certified specialists of the Japan Neurosurgical Society, which may limit generalizability to other institutions. There was no competition for patient care between physicians and NDCs, which could differ in hospitals with more residents or interns. Educational outcomes for trainees were not assessed. In addition, the study’s small sample size, lack of patient outcome measures (e.g., complications or vasospasm rates), and absence of adjustments for potential confounders such as differences in neurosurgeon staffing and unmeasured working hours limit the strength of the findings. These factors underscore the need for larger, controlled studies to validate our observations.

## Conclusions

Introducing an NDC into our neurosurgical department facilitated task shifting in the perioperative management of SAH. Although overall time from ER arrival to ICU/OR admission did not change significantly, NDC involvement contributed to partial redistribution of tasks and reduced the direct burden on neurosurgeons. These findings are consistent with previous studies on JNPs and NPs and extend prior work by providing detailed insight into NDC participation in neurosurgical critical care. Broader implementation of this model may help establish a sustainable clinical treatment system aligned with work-style reforms for doctors in Japan. Broader, multicenter studies are warranted to validate and expand on these findings.
